# The establishment of a universal blocking ELISA for echinococcosis using immunoglobulin against a new conserved linear B-cell epitope

**DOI:** 10.1128/spectrum.03933-25

**Published:** 2026-08-19

**Authors:** Guoyan Zhou, Lijuan Zheng, Zhendong Xin, Jun He, Zhi Li, Hong Duo, Ru Meng, Zhihong Guo, Yong Fu

**Affiliations:** 1 Academy of Animal Science and Veterinary, Qinghai Provincial Key Laboratory of Pathogen Diagnosis for Animal Diseases and Green Technical Research for Prevention and Control, Qinghai University 207475, Xining, China; 2 State Key Laboratory for Diagnosis and Treatment of Severe Zoonotic Infectious Diseases, Key Laboratory for Zoonosis Research of the Ministry of Education, Qinghai University 207475, Xining, China; 3 Key Laboratory of Animal Diseases Diagnostic and Immunology, Ministry of Agriculture, MOE Joint International Research Laboratory of Animal Health and Food Safety, College of Veterinary Medicine, Nanjing Agricultural University 70578, Nanjing, China; 4 Xining Animal Disease Prevention and Control Center, Xining, China; Naturwissenschaftliches und Medizinisches Institut an der Universitat Tubingen, Reutlingen, Germany

**Keywords:** echinococcosis, Em18, monoclonal antibody, B-cell epitope, hybridoma technology

## Abstract

Echinococcosis is a neglected tropical disease that poses a severe threat to global public health and socioeconomic development. Given its alarming global spread, there is an urgent need to develop accurate diagnostic methods to support targeted and personalized treatment. *Echinococcus multilocularis* Em18 has been demonstrated to exhibit the most prominent diagnostic relevance in the progression of alveolar echinococcosis (AE). In this study, six highly specific monoclonal antibodies (mAbs) against the Em18 protein—designated 8G6F, 11E2E, 11E7C, 11E7E, 11E8C, and 11E10D—were generated using hybridoma technology. Indirect ELISA showed that the supernatant titers ranged from 1:1,600 to 1:12,800, while the ascites titers varied from 1:128,000 to 1:512,000. Further analysis indicated that all mAbs belonged to the IgG1 κ isotype. After purification, the ascites-derived mAbs exhibited expected heavy-chain and light-chain bands at approximately 50 kDa and 25 kDa, respectively. Notably, western blot and indirect immunofluorescence assay collectively demonstrated that all mAbs exhibited high specificity for recombinant Em18 protein of approximately 18.65 kDa. Through peptide scanning technology, we identified the antigenic epitope recognized by six mAbs as the region ^99^RMREKHDAKHKS^108^, a highly conserved linear B-cell epitope on the Em18 protein. Amino acid sequence alignment confirmed the complete conservation of this epitope, which is surface-exposed. Furthermore, the optimal blocking antibody 11E8C was selected and labeled with horseradish peroxidase (HRP) based on which a blocking ELISA was established using the anti-Em18 mAbs. Serum samples with a percent inhibition (PI) ≥22.48% were determined as positive, and those with PI ≤17.64% as negative. This method showed no cross-reactivity with positive sera against *Toxoplasma gondii*, *Babesia* spp., *Cysticercus tenuicollis*, *Coenurus cerebralis*, and other non-echinococcosis pathogens. Sensitivity testing showed a positive detection rate of 90%, and positive signals remained stable even when positive sera were diluted up to 1:16. The intra-assay and inter-assay coefficients of variation (CV) were both below 10%. In a concordance evaluation using 47 clinical serum samples from cattle and sheep, the overall agreement between our assay and a commercial kit was 93.62%. Furthermore, the mAbs developed in this study accurately identified intermediate and advanced AE infections, providing a reliable tool for assessing disease progression. Importantly, its cross-reactivity with cystic echinococcosis (CE)-infected sera demonstrates promising potential for the broad-spectrum serodiagnosis of echinococcosis. Collectively, these results suggest that this study provides a novel technical resource for improving serological detection and developing early diagnostic reagents for echinococcosis and offers experimental support for the surveillance, prevention, control, and evaluation of this disease.

**IMPORTANCE:**

Echinococcosis is a neglected tropical disease posing significant threats to global public health and economic development. We have developed six highly specific mAbs capable of precisely recognizing the Em18 protein, a key biomarker for disease progression, and have for the first time identified their antigenic epitope. These antibodies can not only accurately distinguish intermediate and advanced infection stages but also exhibit cross-reactivity with cystic echinococcosis, demonstrating potential as a broad-spectrum diagnostic tool. This achievement paves a new path for developing rapid and precise clinical detection methods, holding significant importance for improving disease prevention and control capabilities.

## INTRODUCTION

As a prevalent but neglected tropical disease (NTD) in the northwestern region of China, alveolar echinococcosis (AE) caused by the metacestode stage of *Echinococcus multilocularis* (*E. multilocularis*, commonly known as the “fox tapeworm”) has been identified as a determinant factor affecting local economic development and animal health (1, 2). Compared to cystic echinococcosis (CE) caused by *Echinococcus granulosus* (*E. granulosus*), AE infection shows more severe pathological changes, clinically presenting as infiltrative tumor-like hepatic lesions with a high potential for dissemination (2). In contrast, neotropical echinococcosis (NE) caused by *Echinococcus vogeli* and *Echinococcus oligarthra* has currently been reported exclusively in South America (3). This geographically constrained distribution serves as a representative case for studying pathogen-geographic correlations in echinococcosis research.

Current evidence indicates that the research history of AE dates back to 1856 when Virchow’s first reported case in Germany marked a milestone in AE research (4, 5). Subsequent case reports have emerged across multiple regions, including Kyrgyzstan, Kazakhstan, and Canada. Of particular concern is current data reveal that AE patients face over 90% mortality within 15 years post-diagnosis (6). Given its substantial health burden, the World Health Organization (WHO) has included echinococcosis in its list of 17 neglected tropical diseases (NTDs) targeted for control or elimination by 2050 (7), underscoring the urgent need to develop effective diagnostic and therapeutic strategies.

Current diagnostic approaches for AE face significant challenges. Imaging faces certain limitations in clinical application due to high equipment costs and technical complexity. In contrast, serological tests have emerged as the preferred diagnostic approach for population screening owing to their advantages such as rapidity and superior specificity. However, their sensitivity and specificity are affected by factors such as parasite species, lesion location, and antigen types (4). Currently, the antigen markers with specific recognition value in the serological diagnosis of AE mainly include Em2, Em492, EmAP, and EmP2 (8), as well as Em10 and its derivatives EmII/3 and Em18 (9). Notably, Em18, a hydrolytic fragment of Em10, has emerged as an excellent diagnostic performance for AE confirmation. Accumulating evidence has demonstrated significant correlations between multiple serological antibody levels and pretreatment parasite lesion-nearby lymph node involvement-metastasis (PNM) stages in AE patients, with anti-Em18 antibodies exhibiting the most prominent diagnostic relevance (4, 9).

Em18, a surface protein in AE lesions, was initially identified as a cleavage fragment of Em10 generated by cysteine protease hydrolysis (10). Recombinant Em18 (rEm18) exhibits outstanding diagnostic potential (11), as western blot analysis demonstrates a sensitivity of 90%–97% (12), comparable to ELISA (sensitivity 92.00%, specificity 96.58%) in large-scale serological testing. Chinese researcher later identified a unique Em18 sequence from Sichuan strains, with its recombinant product demonstrating exceptional diagnostic performance (13). However, two major controversies persist in clinical application, namely cross-reactivity with CE and inter-batch variability in antigen preparation (14). Notably, Swiss patient data revealed that Em18 antibody positivity in CE patients was only 6%–13%, significantly lower than the 82%–92% observed in AE patients (15–17), suggesting that conformational epitopes of Em18 may trigger distinct B-cell responses in AE and CE patients (10). Collectively, these findings highlight the pivotal role of Em18 in echinococcosis. Surprisingly, its antigenic epitopes have yet to be identified, representing a significant gap in our knowledge.

In recent years, the rapid advancement of diagnostic technologies has propelled monoclonal antibody (mAb) research into current focus of concern for researchers, unveiling unprecedented potential for future applications. It plays an irreplaceable role in fields such as disease diagnostics, therapeutic intervention, vaccine development, and targeted drug delivery (18–20). This study generated highly specific mAbs via hybridoma technology and identified their B-cell epitopes, followed by clinical sample validation. Our work provides novel strategies for developing early and rapid diagnostic kits for echinococcosis, as well as innovative approaches for potential vaccine development, thereby contributing to the global efforts in controlling and eliminating this devastating NTD.

## MATERIALS AND METHODS

### Cloning of the Em18 gene

Based on the full-length Em18 gene sequence of *E. multilocularis* (GenBank: AY513691) from the NCBI database, we introduced *BamH*I and *Xho*I restriction sites flanked by protective bases at the 5′ and 3′ termini, respectively. The target gene was synthesized by Tsingke Biotechnology Co., Ltd. (Nanjing, China). For the polymerase chain reaction (PCR) amplification, specific primers were designed (Forward: 5′-ATGAAGGAGTCTGACTTAGCGGATATGAAG-3′ and Reverse: 5′-CTATTTGAGGTTGGCCAGCTTCGTCTG-3′). The reaction conditions involved initial denaturation at 95°C for 5 min, followed by 35 cycles of denaturation (95°C, 30 s), annealing (63°C, 30 s), and extension (72°C, 90 s), with a final extension at 72°C for 5 min. The PCR amplified product was electrophoresed on a 1% agarose gel and subsequently purified using the MolPure Fast Gel Extraction Kit (Yeasen Biotechnology, Shanghai) for fragment recovery.

### Construction of recombinant pET-28a-Em18 plasmid

Following the manufacturer’s protocol, we directionally ligated the target fragment into the linearized pET-28a vector using the Beyotime Seamless Cloning Kit to generate the recombinant plasmid pET-28a-Em18 with an N-terminal His-tag. The ligation construct was transformed into *E. coli* DH5α competent cells via an optimized transformation involving 30 min on ice, 90 s at 42°C, followed by 3 min on ice. Subsequently, transformed cells were plated on Luria-Bertani (LB) agar plates containing kanamycin and incubated overnight at 37°C. Positive colonies were inoculated into LB liquid medium with kanamycin and cultured with shaking (220 rpm) at 37°C for approximately 12 h. Finally, PCR-positive clones were subsequently verified by Sanger sequencing (Sangon Biotech, Shanghai), and confirmed plasmids were stored at −80°C for long-term preservation.

### Optimization of expression conditions and purification of rEm18 protein

The recombinant plasmid pET-28a-Em18 was transformed into *E. coli* BL21(DE3) competent cells to express the His-tagged fusion protein. To optimize protein expression, we tested key induction parameters including temperature (16°C, 25°C, and 37°C), incubation time (12–20 h), and isopropyl β-D-1-thiogalactopyranoside (IPTG) concentration (0.2–1.0 mmol/L). Protein expression was induced with IPTG when cultures reached mid-log phase (OD600 = 0.6–0.8). After harvesting, cells were lysed by ultrasonication, and samples (whole-cell lysate, supernatant, and pellet) were collected for analysis. The target protein was purified using Ni-NTA affinity chromatography (GenScript Biotech). Nonspecific proteins were initially washed with low-concentration imidazole buffer (40 mmol/L) and followed by specific elution of target proteins with high-concentration imidazole buffer (100–500 mmol/L). Subsequently, protein expression and purity were assessed via 12% sodium dodecyl sulfate-polyacrylamide gel electrophoresis (SDS-PAGE) with Coomassie-blue staining.

### Determination of purified protein concentration

Protein concentration was quantified using a BCA Protein Assay Kit (Yisheng Biotechnology Co., Ltd., Shanghai, China). The detailed procedure involved freshly prepared BCA working solution by mixing Reagent A and Reagent B at a volume ratio of 50:1 in a 50 mL centrifuge tube. Protein standard solutions were pipetted into 1.5 mL centrifuge tubes at volumes of 0, 0.8, 4, 8, 12, 20, 24, 28, and 40 μL, respectively, and each tube was supplemented with phosphate-buffered saline (PBS) to a final volume of 40 μL. Subsequently, 10 μL of each standard dilution and test samples was transferred to a 96-well plate in triplicate replicates. Then, 200 μL of the prepared BCA working solution was added to each well, followed by thorough mixing via gentle pipetting. The plate was incubated at 37°C for 30 min, and the absorbance at 562 nm was measured. A standard curve was constructed to calculate the protein concentration of the test samples. The purified protein was aliquoted and stored at −80°C.

### Mice immunization protocol and indirect ELISA

Female BALB/c mice (*n* = 5, 6–8 weeks old) were subcutaneously immunized with 100 μg rEm18 protein emulsified with Freund’s adjuvant at a 1:1 volume ratio via multiple injections on the dorsal neck region. Complete Freund’s adjuvant was used for the primary immunization, while incomplete Freund’s adjuvant was applied for booster immunizations. Serum samples were collected 1 week after the third immunization from the tail vein, and antibody titers were determined by iELISA. A negative control group (*n* = 1) received PBS emulsified with adjuvant following the same immunization schedule.

The iELISA was adapted as previously described (21). Briefly, rEm18 protein was diluted to 2 μg/mL in carbonate-bicarbonate coating buffer (pH 9.6) and coated (100 μL/well) into 96-well microplates for overnight incubation at 4°C. After washing with PBS containing 0.05% Tween-20 (PBST), plates were blocked with 5% skimmed milk (100 μL/well) in PBST for 1 h at 37°C. Subsequently, serum samples of immunized mice (primary antibody, 1:100 dilution) were added (100 μL/well) and incubated for 1 h at 37°C. Following three washes, 100 μL of HRP-conjugated goat anti-mouse IgG (H + L) (secondary antibody, 1:3,000 dilution) was added to each well and incubated at 37°C for 1 h. Then, enzymatic activity was detected by adding 100 μL/well of 3,3′,5,5′-tetramethylbenzidine (TMB) substrate for 10–15 min reaction at 37°C in the dark, and the reaction was stopped with 50 μL of 2 M sulfuric acid (H₂SO₄). Finally, the absorbance was measured at 450 nm using a Tecan M200 Pro multi-mode microplate reader.

### Establishment of positive hybridoma cell lines

SP2/0 cells in optimal growth phase were prepared for cell fusion. One day prior to fusion, peritoneal cells were aseptically isolated from a naïve BALB/c mouse to prepare feeder layers, which were subsequently seeded into 96-well culture plates. Immunized BALB/c mice exhibiting high antibody titers (≥1:10^4^) received a final booster immunization. Three days later, splenocytes were isolated and mixed with SP2/0 cells at a 7:1 ratio, followed by centrifugation and supernatant removal. Polyethylene glycol (PEG) fusion agent was gradually added to the cell pellet, with subsequent stepwise addition of medium to terminate fusion. The fused cells were centrifuged, resuspended, and distributed into the feeder layer-containing 96-well plates for culture at 37°C with 5% CO₂.

After 7–10 days of culture, cell supernatants were collected as primary antibodies for initial screening via iELISA. Wells exhibiting a P/N ratio ≥2.1 were considered positive. Positive clones were subsequently subcloned by limiting dilution, adjusting cell density to approximately 1 cell/0.1 mL before seeding onto feeder layer plates. Cells were regularly monitored and tested, ultimately establishing stable hybridoma cell lines capable of consistent mAb secretion.

For *in vivo* antibody production, six 6- 8-week-old female BALB/c mice were intraperitoneally injected with 500 μL of liquid paraffin for priming. Seven days later, each mouse received a single intraperitoneal injection of 2 × 10^6^ positive hybridoma cells (one cell line per mouse). Mouse conditions were monitored daily. Upon significant abdominal distension (typically after 12–14 days), ascites fluid was collected. The ascites was centrifuged at 5,000 rpm for 10 min at 4°C, and the supernatant was harvested for determination of antibody titers using iELISA.

### Characterization of mAbs

To systematically characterize the obtained mAbs, isotype determination was conducted using a Mouse Monoclonal Antibody Isotyping Kit (Proteintech), which precisely identified both heavy chain (IgG1/IgG2a/IgG2b/IgG2c/IgG3/IgM) and light chain (kappa/lambda). Purification used Protein G Agarose (smart-lifesciences) with glycine-HCl elution (pH 2.7). Additionally, we quantitatively determined the antibody titer of mAbs using iELISA. Ultimately, the protein concentration of the purified mAbs was accurately quantified via the BCA assay.

### Specificity detection by western blot and indirect immunofluorescence assay

To systematically characterize the specificity of the six mAbs, we employed a dual-validation system combining western blot and IFA. REm18 was separated by 12% SDS-PAGE and transferred to PolyVinylideneFluoride (PVDF) membranes using a semi-dry transfer system (23 V, 33 min). Membranes were blocked with 5% skimmed milk in Tris-buffered saline containing 0.05% Tween-20 (TBST) for 1 h at room temperature. Membranes were then incubated with mAbs (1:5,000 dilution) overnight at 4°C, followed by incubation with HRP-conjugated goat anti-mouse IgG (H + L) (secondary antibody, 1:3,000 dilution) for 1 h at 37°C. Finally, signals were detected using Super ECL Detection Reagent (Yeasen Biotechnology) and imaged with a Tanon chemiluminescence imaging system.

The binding ability of six mAbs against *E. multilocularis* protoscoleces was evaluated by IFA. Protoscolex samples were collected from hepatic cysts of C57BL/6 mice intraperitoneally infected with *E. multilocularis* for 6 months in our laboratory, the infected mice were anesthetized and sacrificed by cervical dislocation followed by immersion in 75% ethanol for 10 min, the cyst tissues were isolated in a biological safety cabinet and immersed in 0.9% normal saline, the cysts were cut into pieces to fully release protoscoleces which were then filtered through 100 μm and 40 μm meshes and collected in centrifuge tubes, washed with 0.9% normal saline until the supernatant became clear, and their viability was assessed by trypan blue staining; nonspecific binding sites were blocked with 5% bovine serum albumin (BSA) for 2 h at room temperature, followed by overnight incubation at 4°C with six purified mAbs (1:3,000 dilution). After PBST washing, Alexa Fluor 594 goat anti-mouse IgG (H + L) as the secondary antibody (1:3,000 dilution) was applied and incubated for 1 h at 37°C in the dark. Unbound antibodies were removed by extensive PBST washing, fluorescence signals were visualized and captured using an inverted fluorescence microscope (Model MIX60, Guangzhou Mingmei Optoelectronic Technology Co., Ltd., China).

### Identification of linear B-cell epitopes in rEm18 protein

To comprehensively elucidate the linear B-cell epitopes of the Em18 antigen, we employed a comprehensive epitope mapping strategy using truncated peptide scanning. A series of gene-specific primers flanked by *EcoR*I and *Xho*I restriction sites were designed according to the codon-optimized Em18 sequence (Table 1). These primers enabled the successful amplification of various truncated Em18 fragments using the pET-28a-Em18 recombinant plasmid as template. The pET-32a (+) expression vector was subsequently digested with *EcoR*I and *Xho*I restriction enzymes, and the amplified truncation fragments were precisely ligated into the linearized vector using seamless cloning technology. The resulting constructs were transformed into *E. coli* DH5α competent cells, followed by systematic screening of single colonies through colony PCR. Positive clones containing inserts of correct size were validated by sequencing. The sequence-verified recombinant plasmids were amplified, purified, and subsequently transformed into *E. coli* BL21(DE3) expression strains for protein induction, with empty pET-32a (+) vector serving as negative control. Following induction, bacterial cells were lysed by ultrasonication and subjected to heat-denaturation treatment. Protein expression was confirmed by western blot analysis using anti-His tag primary antibody and HRP-conjugated goat anti-mouse IgG secondary antibody. Finally, the successfully expressed truncation proteins served as detection antigens. Six mAbs were employed as primary antibodies to probe against these truncation variants, enabling precise identification of the linear B-cell epitopes recognized by each mAb. Subsequently, the three-dimensional structure of Em18 was modeled using AlphaFold3. This high-confidence structural prediction was subsequently visualized in PyMol, allowing us to precisely map and illustrate the spatial arrangement of the predicted epitopes within their native three-dimensional context.

**TABLE 1:** Primers sequence for truncated Em18 protein

Primer	Amplified region	Sequence (5′−3′)
Em18-F1 F	1–64 aa	GGCTGATATCGGATCCGAATTCATGAAAGAATCAGACCTAGCTGATAT
Em18-F1 R		GGTGGTGGTGGTGCTCGAGGCGGTTGC
Em18-F2 F	54–117 aa	GATATCGGATCCGAATTCACGGCCGCGTCTGCGGAGG
Em18-F2 R		GTGGTGGTGGTGCTCGAGATCACCGCTGCCATTTACC
Em18-F3 F	107–161 aa	TGATATCGGATCCGAATTCCACAAAAGCCAGGTAAATGGC
Em18-F3 R		GGTGGTGGTGGTGCTCGAGCTTCAGATTAGCCAATTTGGTTTG
Em18-F4 F	65–98 aa	GGCTGATATCGGATCCGAATTCATGCTGATGGCTCAGAGAGA
Em18-F4 R		GGTGGTGCTCGAGCTCCGCCTCTGCCTGC
Em18-F5 F	99–132 aa	GATATCGGATCCGAATTCCTGCGTCGTATGCGTGAGAAG
Em18-F5 R		GGTGGTGGTGCTCGAGCTCCTTCGCTTCGGAC
Em18-F6 F	133–161 aa	GATATCGGATCCGAATTCTTGGAAGTGATTCCGAACGTG
Em18-F6 R		GGTGGTGGTGGTGCTCGAGCTTCAGATTAGCCAATTTG
Em18-F7 F	65–108 aa	GATATCGGATCCGAATTCATGCTGATGGCTCAGAGAGAT
Em18-F7 R		GGTGGTGGTGCTCGAGCGCATCGTGCTTCTCA
Em18-F8 F	65–118 aa	GATATCGGATCCGAATTCATGCTGATGGCTCAGAGAGAT
Em18-F8 R		GGTGGTGGTGCTCGAGACCGCTGCCATTTACCT

### Establishment of a blocking ELISA and screening of the optimal blocking antibody

The Em18 protein was diluted to 4 μg/mL using carbonate coating buffer, and 100 μL of the solution was added to each well of a 96-well microplate for overnight coating at 4°C. The plate was then washed three times with PBST and tapped dry to remove residual liquid. Subsequently, 100 μL of 3% skimmed milk was added to each well, followed by blocking on a shaker at 37°C for 1 h. Negative and positive control sera were diluted at a ratio of 1:10, added to individual wells at 100 μL per well, and incubated at 37°C for 1 h. MAbs diluted at 1:3,000 and HRP-conjugated goat anti-mouse IgG diluted at 1:3,000 were successively added at 100 μL per well, with both incubation steps performed at 37°C for 1 h. Thereafter, 100 μL of TMB chromogenic solution was added to each well for color development in the dark for 10–15 min, and the reaction was terminated by the addition of 50 μL TMB stop solution. Finally, absorbance values were measured at 450 nm using a multifunctional microplate reader, and the percent inhibition (PI) was calculated accordingly (22).


$$PI\;(\%)=\frac{OD450\;nm\;(\text{negative})-OD450\;nm\;(\text{test})}{OD450\;nm\;(\text{negative})}\times 100\%$$

### HRP labeling of the optimal blocking antibody

The optimal blocking antibody identified in the screening process was labeled using an HRP rapid labeling kit following a refined protocol. Briefly, the purified antibody was first subjected to ultrafiltration in 0.01 M carbonate buffer (CB) to eliminate interfering substances and ensure efficient labeling efficiency. Then, 500 μL of the purified antibody was mixed with 50 μL of pre-activated HRP solution by gentle and repeated pipetting, followed by the addition of 99 μL HRP labeling initiator at a ratio of 1.8 μL initiator per 10 μL antibody-HRP mixture, and the mixture was gently blended several times to avoid bubble formation before incubation in a 37°C incubator in the dark for 2–3 h to facilitate conjugation. Meanwhile, 1 mL of double-distilled water was added to the lyophilized reaction stop solution and mixed thoroughly, and 50 μL of the reconstituted stop solution was introduced into the above reaction system at a ratio of 50 μL stop solution per 1 mg HRP, followed by thorough mixing and incubation at room temperature for 1 h. Finally, an equal volume of labeling stabilizer was added, and the mixture was blended and stored at −20°C.

In subsequent blocking ELISA assays, the sequential addition of mAbs and HRP-conjugated goat anti-mouse IgG as described in the establishment of the blocking ELISA was simplified to a single step: HRP-11E8C was diluted at 1:3,000 in PBS, added to 96-well microplates at 100 μL per well, and incubated at 37°C for 1 h.

### Determination of the cut-off value

A total of 45 serum samples confirmed negative by a commercial Echinococcosis ELISA antibody detection kit were subjected to PI measurement, from which the mean PI value (X) and standard deviation (SD) were calculated. Samples with a PI value ≥X+ 3 SD were defined as positive, those with PI ≤X+ 2 SD were classified as negative, and samples with PI values falling between these two thresholds were regarded as suspect.

### Methodological evaluation of the established blocking ELISA

#### Specificity assay

Positive sera against common non-*Echinococcus* pathogens prevalent in cattle and sheep, including *Toxoplasma gondii*, *Babesia* spp., *Theileria annulata*, *Balantidium coli*, *Cysticercus tenuicollis,* and *Coenurus cerebralis*, were collected from laboratory stocks and subjected to PI determination. Positive sera against *E. granulosus* and *E. multilocularis* were included as controls, and the serological status was judged based on the calculated PI values.

#### Sensitivity assay

Thirty serum samples confirmed positive for echinococcosis using a commercial Eg95-based detection kit were tested to calculate the positive detection rate for the assessment of the diagnostic sensitivity. Additionally, three positive hydatid disease sera were serially diluted with PBS at ratios of neat, 1:1, 1:2, 1:4, 1:8, and 1:16, the PI was determined for each dilution. The maximum dilution factor that still judged positive was regarded as the analytical sensitivity of the established method.

#### Repeatability assay

Intra-assay repeatability was evaluated using purified Em18 protein from the same batch as the coating antigen. Three positive and three negative hydatid disease serum samples were each tested in eight replicates, and the coefficient of variation (CV) was calculated. For inter-assay repeatability, the same batch of purified Em18 protein was coated on five separate ELISA plates and tested over 3 consecutive days using the same panel of positive and negative sera; the CV was calculated to assess the consistency of the assay across different plates and time points (22).


$$CV\;(\%)=\frac{SD}{\bar{X}}\times 100\%$$

#### Concordance assay

A blocking ELISA was used to detect 47 clinical sheep serum samples, and the results were compared with those obtained using the Sheep Hydatid Cyst Eg95 Detection Kit (Shenzhen Kangbaide Biotechnology Co., Ltd.). The concordance rate was calculated according to the following formula (23):


$$Concordance\;rate\;(\%)=\frac{Number\;of\;positive\;samples\;+\;Number\;of\;negative\;samples}{Total\;number\;of\;tested\;samples}\times 100\%$$

### Serological detection

The blocking ELISA established in this study was utilized to evaluate its detection performance for sera at different stages of *E. multilocularis* infection. Serum samples were collected from C57BL/6J wild-type mice infected with *E. multilocularis* (*n* = 3 per group) at critical time points post-infection (1, 2, and 6 months), with sera from uninfected mice included as negative controls. The serological response profiles at different infection stages were systematically analyzed.

## RESULTS

### Target gene cloning and recombinant plasmid construction

**Fig 1:**
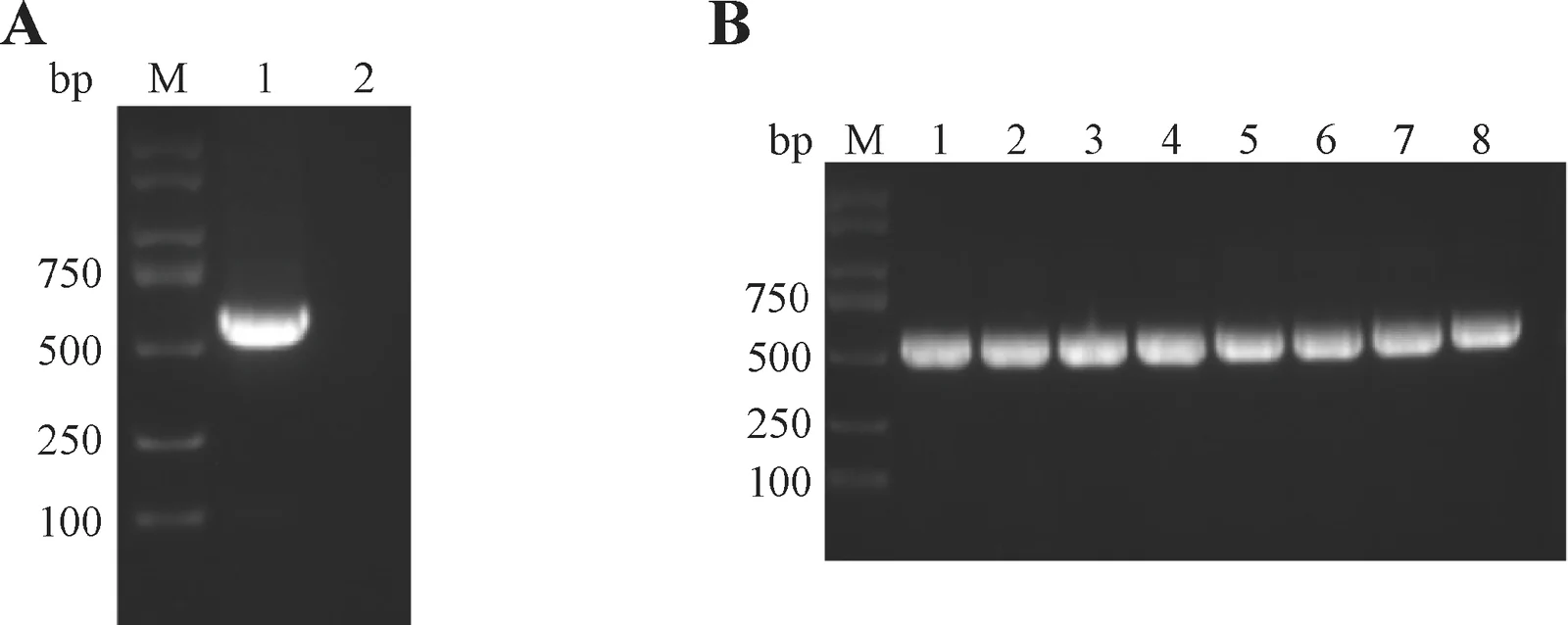
Molecular validation of Em18 gene cloning and recombinant plasmid construction. (**A**) PCR amplification of the *E. multilocularis* Em18 gene. Lane M: DNA ladder; Lane 1: PCR product of Em18 gene; Lane 2: Negative control. (**B**) PCR identification of bacterial fluids. Lane M: DNA ladder; Lanes 1–8: PCR products from randomly selected transformants.

To generate rEm18 antigen for immunization, the target gene was amplified by PCR using specific primers. Electrophoresis on a 1% agarose gel showed a clear 486 bp band, corresponding to the expected size of Em18 (Fig. 1A). The purified PCR product was subsequently ligated into the pET-28a vector and transformed into DH5α competent cells, and randomly selected single colonies were subjected to PCR validation, which successfully amplified fragments of 486 bp (Fig. 1B). Sanger sequencing (Sangon Biotech) demonstrated 100% identity between the cloned fragment and the reference Em18 gene, validating the correct construction of pET-28a-Em18.

### Optimization of rEm18 expression and purification

The verified pET-28a-Em18 recombinant plasmid was transformed into BL21(DE3) competent cells for induced expression. After comprehensive evaluation, the optimal expression conditions were obtained with 0.4 mM IPTG induction at 16°C for 20 h. Subsequently, the rEm18 protein was efficiently purified. SDS-PAGE analysis demonstrated a predominant band at 18.65 kDa with a purity of more than 90% (Fig. S1), confirming successful purification. The purified Em18 protein was used for immunization and mAbs generation.

### Determination of purified protein concentration

The concentration of the purified protein was determined using a BCA protein assay kit, with the absorbance measured at 562 nm was 0.3154. Based on the standard curve equation (Fig. S2), which showed a regression coefficient *R*² > 0.99, the concentration of the rEm18 protein was calculated to be 0.584 mg/mL.

### Hybridoma generation and mAbs screening

**Fig 2:**
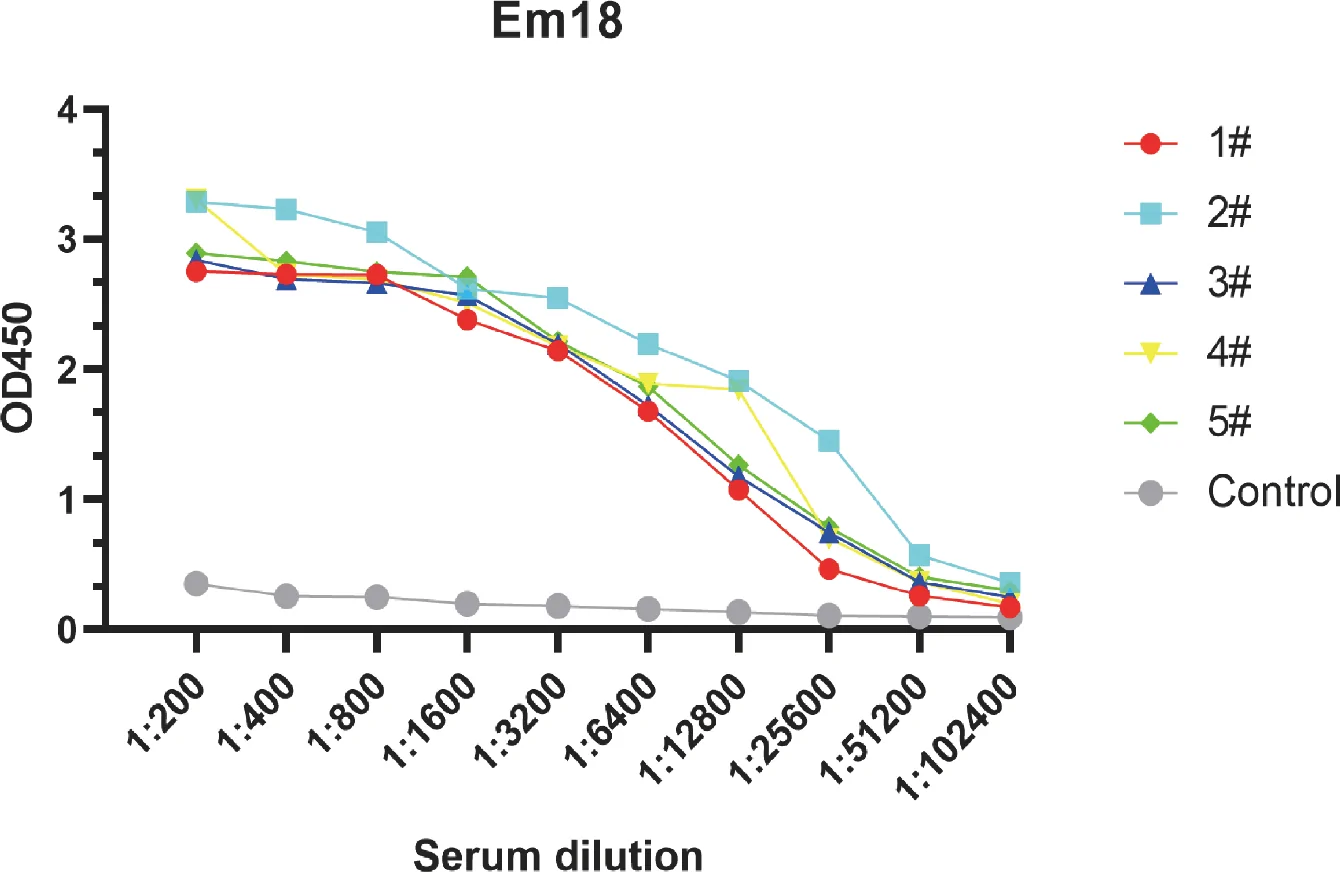
Evaluation of serum antibody titer. Dynamic changes in serum antibody titers of mice immunized with recombinant protein. Serum from BALB/c mice (*n* = 5, color-coded) immunized three times with the recombinant protein showed progressively increasing IgG antibody titers, indicating robust immunogenicity. Control group (gray) confirms specificity.

Following three immunization cycles of purified Em18 protein at 2-week intervals, BALB/c mice exhibited high anti-Em18 antibody titers. The antibody titer was determined by iELISA as the highest serum dilution yielding a *P*/*N* ≥ 2.1 (*P*-value: immunized sera; *N*-value: non-immunized sera), and the serum titers reached >1:10^4^ (Fig. 2). Hybridomas were selected in HAT medium, and through three to four rounds of limiting dilution cloning and rEm18-specific ELISA screening, six stable hybridoma cells were established, and the cells demonstrated robust growth characteristics. These cells were named 8G6F, 11E2E, 11E7C, 11E7E, 11E8C, and 11E10D.

### Characterization of six anti-Em18 mAbs

**Fig 3:**
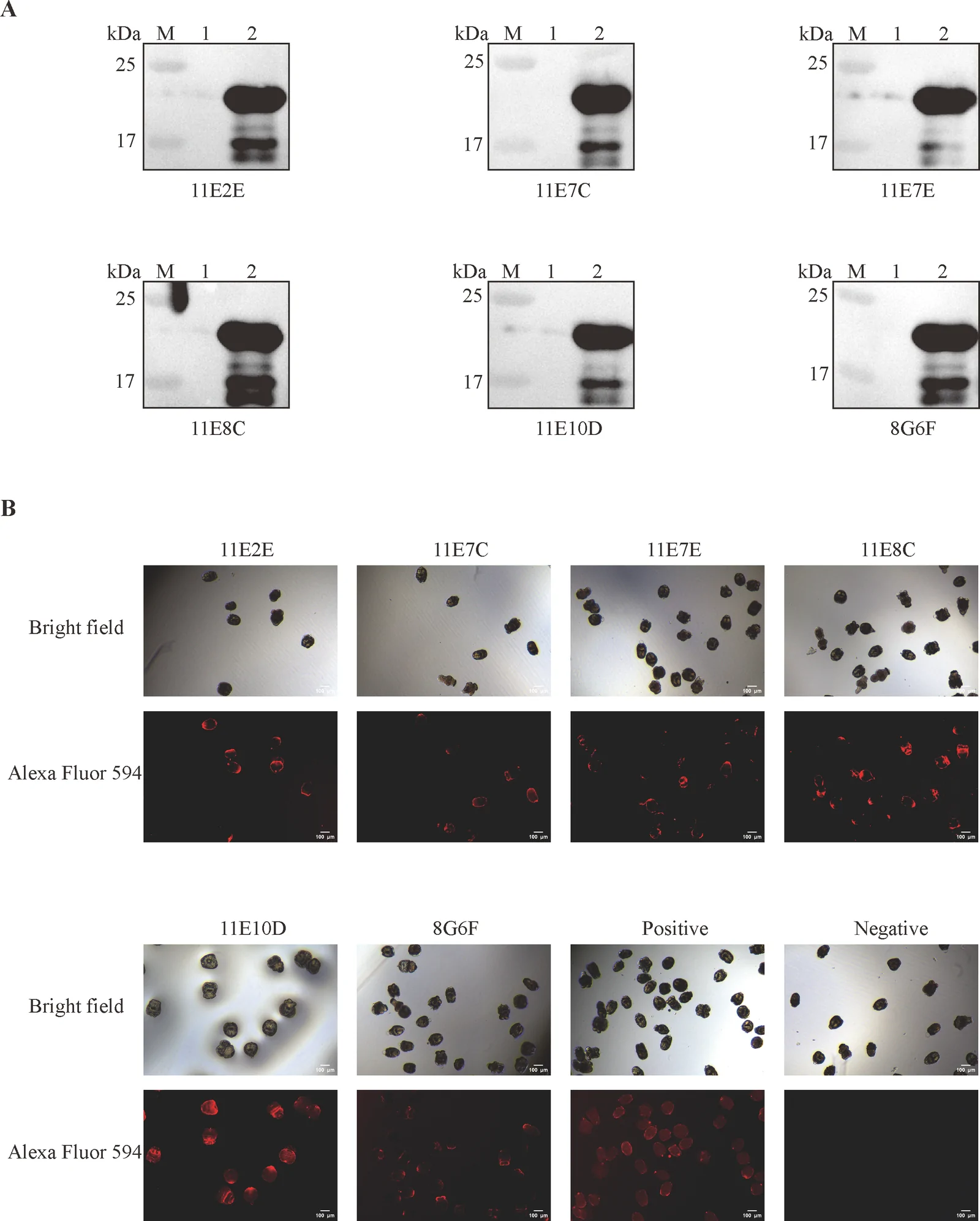
Evaluation of the specificity of mAbs against Em18 protein. (**A**) The western blot was incubated with mAbs 11E2E, 11E7C, 11E7E, 11E8C, 11E10D, and 8G6F, respectively. Lane M: Protein molecular weight marker; Lane 1: pET28a-BL21(DE3) bacterial lysate; Lane 2: Purified Em18 protein. (**B**) IFA identification of monoclonal antibody. The binding specificity of six mAbs (11E2E, 11E7C, 11E7E, 11E8C, 11E10D, 8G6F) against *E. multilocularis* protoscoleces was evaluated via IFA. Negative and positive control sera were included, and Alexa Fluor 594-conjugated goat anti-mouse IgG (H + L) was used as the secondary antibody. Fluorescent signals were visualized under an inverted microscope at 400× magnification. Scale bars 100 μm.

Western blot analysis confirmed that all six mAbs specifically recognized the target Em18 antigen, showing strong reactivity at the 18.65 kDa band corresponding to rEm18 (Fig. 3A). Remarkably, all six prepared mAbs demonstrated intense red fluorescence signals specifically binding to protoscoleces, while no fluorescent signal was detected in negative controls (Fig. 3B). Isotype characterization revealed that these mAbs belonged to the IgG1 subclass with kappa light chains (Table 2). Importantly, SDS-PAGE analysis of the purified antibodies further demonstrated their expected molecular weights, with heavy chains at 50 kDa and light chains at 25 kDa (Fig. S3).

**TABLE 2:** Isotypes of six hybridoma-derived mAbs against Em18 proteins (OD450)^*a*^

mAbs	Isotypes								
	mAb subclass						Light chains		
	IgG1	IgG2a	IgG2b	IgG2c	IgG3	IgM	Kappa		Lambda
11E2E	**2.7235**	0.6425	0.1348	0.0838	0.0792	0.1522	**1.3086**		0.0793
11E7C	**2.7645**	0.2507	0.3703	0.0774	0.0809	0.4159	**1.4399**		0.0810
11E7E	**2.7926**	0.2360	0.1391	0.0734	0.0764	0.2041	**1.2983**		0.0787
11E8C	**2.7194**	0.2200	0.1186	0.0789	0.0745	0.0832	**1.1584**		0.0796
11E10D	**2.8719**	0.2957	0.1835	0.0835	0.0813	0.2213	**1.3395**		0.0860
8G6F	**2.8048**	1.0212	0.3840	0.0728	0.0752	0.0914	**1.6271**		0.0802

^*a*^ Bold values represent the highest values within each row, highlighting the most prominent result for each group.

The ascites titers were determined by iELISA and ranged from 6 × 10^4^ to 2 × 10^5^, the antibody titers in ascites varied from 1:64,000 to 1:256,000 (Table S1), where the maximum serum dilution yielding a *P*/*N* ratio ≥2.1 was defined as the final titer. Additionally, spectrophotometric analysis confirmed high antibody concentrations (0.55–4.37 mg/mL), ensuring sufficient quantities for downstream applications.

### Identification of linear B-cell epitopes in rEm18 protein

**Fig 4:**
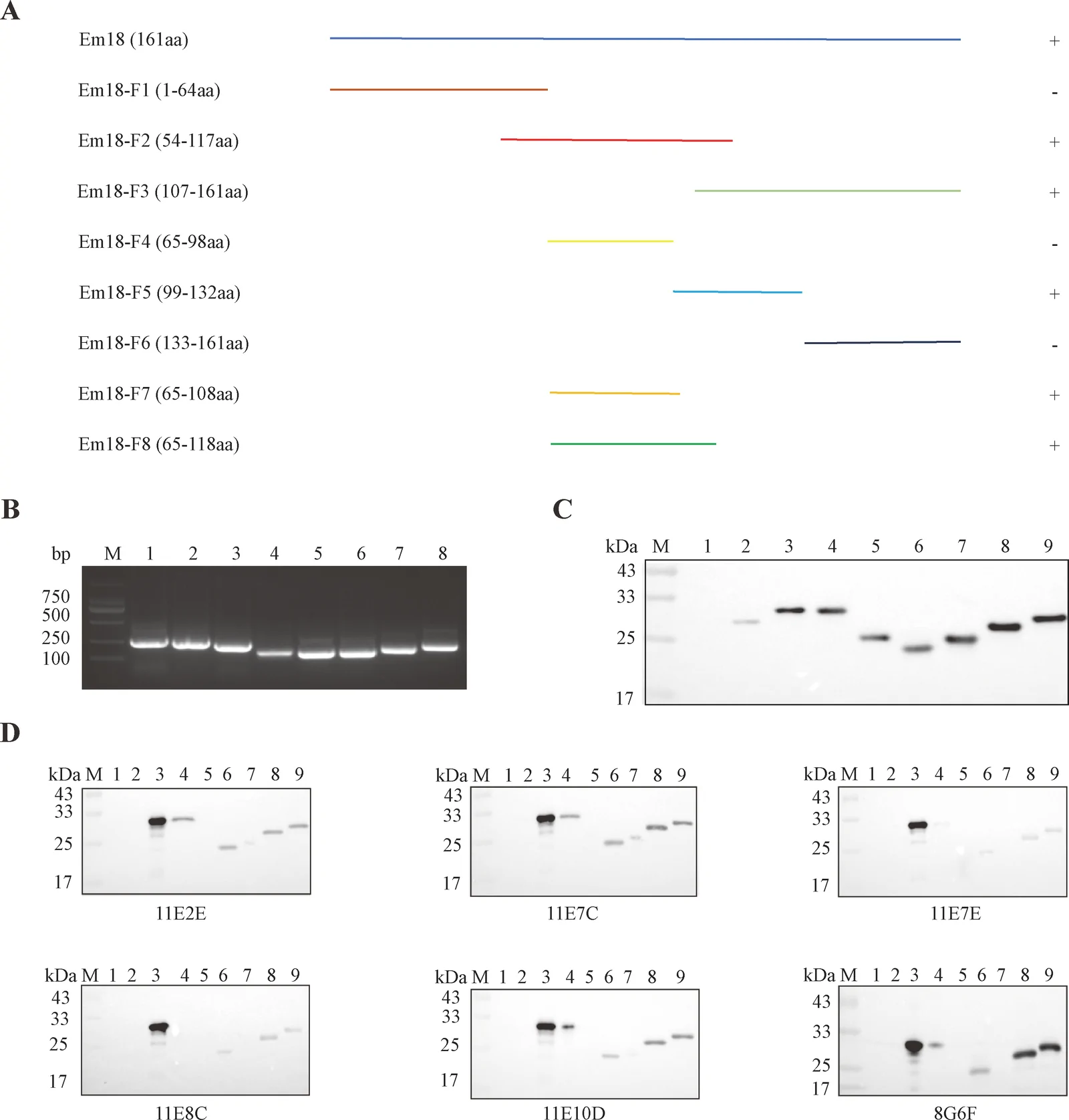
Identification of linear B-cell epitopes in rEm18 protein. (**A**) Mapping strategy for B-cell epitopes recognized by mAbs using truncated fragments. Eight overlapping truncated Em18 fragments (Em18-F1 to Em18-F8) expressed in *E. coli* BL21. (**B**) PCR amplification of eight truncated fragments of the Em18 gene. M: DL 2000 DNA Marker; 1: Em18-F1 amplification product; 2: Em18-F2 amplification product; 3: Em18-F3 amplification product; 4: Em18-F4 amplification product; 5: Em18-F5 amplification product; 6: Em18-F6 amplification product; 7: Em18-F7 amplification product; 8: Em18-F8 amplification product. (**C**) Expression validation of Em18 truncation mutants. Lane M: Protein molecular weight marker; Lane 1: pET-32a empty vector; Lanes 2-9: Em18-F1, Em18-F2, Em18-F3, Em18-F4, Em18-F5, Em18-F6, Em18-F7, and Em18-F8, respectively. (**D**) Identification of Em18 mAbs epitopes. Lane M: Protein molecular weight marker; Lane 1: pET-32a empty vector; Lanes 2–9: Em18-F1, Em18-F2, Em18-F3, Em18-F4, Em18-F5, Em18-F6, Em18-F7, and Em18-F8, respectively.

To precisely identify the antigenic epitopes recognized by Em18 mAbs, we systematically constructed eight recombinant truncated proteins of Em18 using a comprehensive peptide scanning approach (Fig. 4A). PCR amplification of the truncated gene fragments yielded products that were separated by 1% nucleic acid gel electrophoresis (Fig. 4B), all target gene fragments exhibited the expected molecular sizes, confirming successful amplification. Western blot analysis using anti-His tag antibody as the primary antibody was performed to verify plasmid expression. The results demonstrated specific bands at predicted molecular weights for all truncated constructs (Fig. 4C), validating successful protein expression. Finally, epitope mapping through western blot analysis revealed the reactivity patterns of six mAbs against the truncated protein panel (Fig. 4D), all six mAbs showed positive reactivity with fragments F2, F3, F5, F7, and F8, while demonstrating no reactivity with F1, F4, and F6.

**Fig 5:**
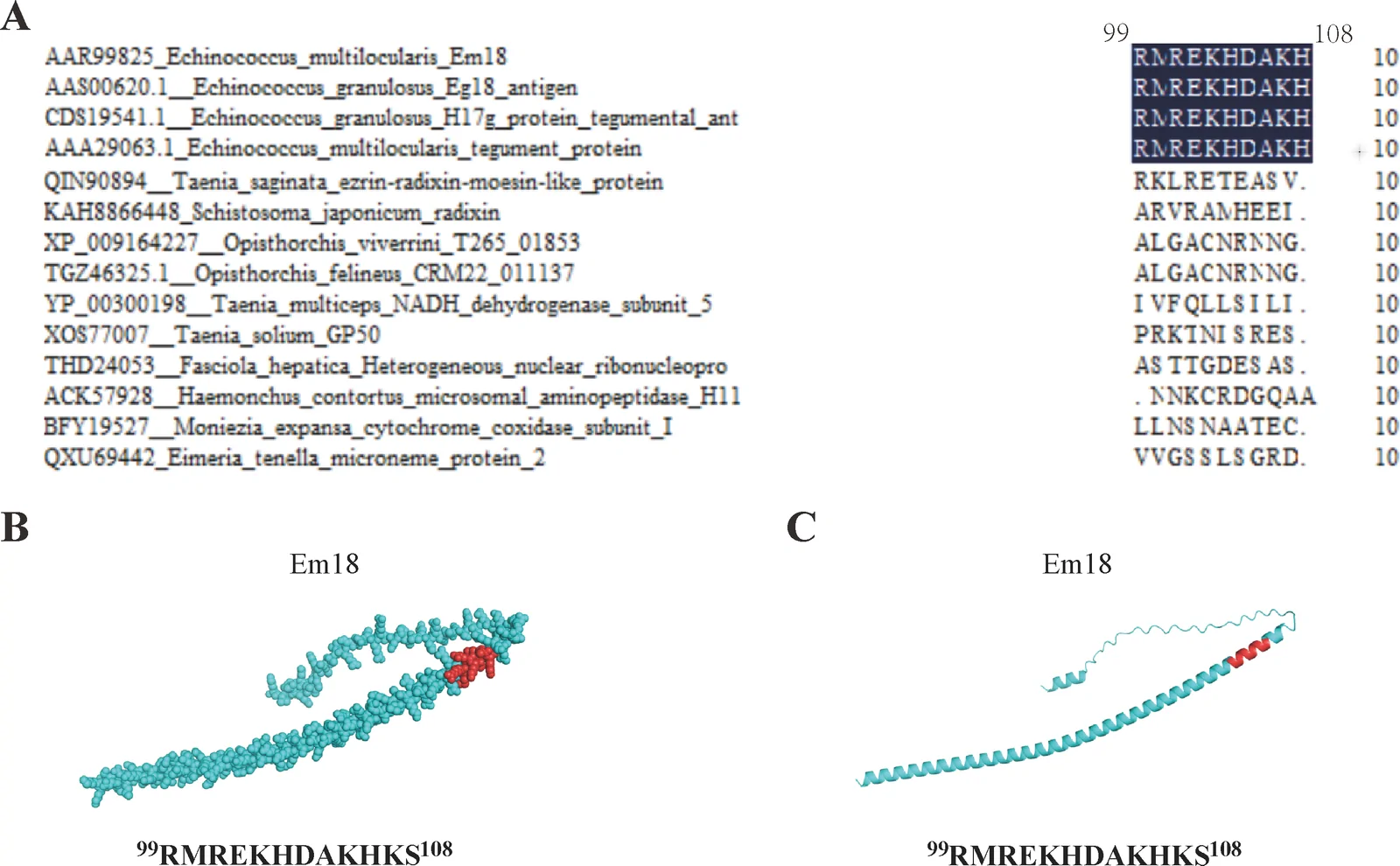
Sequence alignment and spatial structure analysis of the identified epitope on Em18 protein. (**A**) Sequence alignment of the ^99^RMREKHDAKHKS^108^ epitope across different species; (**B and C**) the epitope was highlighted in red within the simulated three-dimensional structure of the Em18 protein using PyMol software and presented as a spherical model (**B**) and a cartoon model (**C**), respectively.

The results demonstrate that all six Em18 mAbs target the linear epitope within the ^99^RMREKHDAKHKS^108^ domain. Amino acid sequence alignment confirms the high conservation of this epitope across *Echinococcus* species (e.g., *E. granulosus*, E. multilocularis), while revealing its absence in related taeniid cestodes and common livestock parasites such as *Taeniidae* and *Fasciola hepatica*. This pattern underscores the epitope’s specificity to *Echinococcus* and indicates a low risk of cross-reactivity, thereby ensuring diagnostic specificity (Fig. 5A). Notably, the epitope is exposed on the Em18 surface as a prominent α-helix (Fig. 5B and C), suggesting high spatial accessibility and positioning it as a highly promising antigenic site.

### Screening of the optimal blocking antibody

The blocking efficacy of six mAbs was evaluated using the established blocking ELISA (Table S2). The mAbs were ranked in descending order of PI as follows: 11E8C, 11E7C, 8G6F, 11E10D, 11E7E, and 11E2E. The mAb with the highest PI value (79.8%) was selected as the optimal blocking antibody, namely 11E8C.

### Determination of the cut-off value

The test results of 45 clinical negative serum samples are shown in Table 3. The X was 7.97% and the SD was 4.84%, corresponding to X+ 2 SD = 17.65% and X+ 3 SD = 22.49%. Serum samples with a PI value ≥22.49% were judged positive for echinococcosis antibodies, those with a PI value ≤17.65% were considered negative, and samples with PI values between 17.65% and 22.49% were classified as suspicious and required retesting.

**TABLE 3:** Determination of the cut-off value

Serum ID	PI (%)	Serum ID	PI (%)	Serum ID	PI (%)
1#	6.01	16#	4.77	31#	13.42
2#	4.31	17#	9.62	32#	1.21
3#	15.94	18#	15.12	33#	6.82
4#	7.67	19#	9.28	34#	4.06
5#	3.27	20#	9.19	35#	3.67
6#	5.81	21#	5.82	36#	12.91
7#	15.46	22#	6.43	37#	3.19
8#	1.20	23#	9.70	38#	4.29
9#	2.93	24#	2.96	39#	13.93
10#	15.88	25#	4.94	40#	6.32
11#	6.29	26#	14.97	41#	9.98
12#	9.50	27#	16.55	42#	3.24
13#	3.62	28#	17.15	43#	1.78
14#	10.43	29#	10.46	44#	3.04
15#	6.07	30#	3.82	45#	15.57

### Methodological evaluation of blocking ELISA

#### Specificity assay

Seven non-*Echinococcus* positive serum samples and six echinococcosis-positive serum samples stored in the laboratory were tested (Table 4), all seven non-*Echinococcus* sera tested negative, whereas all six echinococcosis sera tested positive, indicating excellent specificity of the established method.

**TABLE 4:** Specificity test results of blocking ELISA

Serum samples	PI (%)	Test result^*a*^
*Toxoplasma gondii*	10.32	−
*Babesia bovis*	2.88	−
*Babesia motasi*	7.82	−
*Theileria annulata*	2.23	**−**
*Balantidium coli*	5.65	**−**
*Cysticercus tenuicollis*	4.16	**−**
*Coenurus cerebralis*	3.48	**−**
*E. granulosus* 1	53.52	+
*E. granulosus* 2	42.86	+
*E. granulosus* 3	55.03	+
*E. multilocularis* 1	30.98	+
*E. multilocularis* 2	29.77	+
*E. multilocularis* 3	31.27	+

^*a*^ “−” and “+” indicate negative and positive results, respectively, for the presence of the target antibody.

#### Sensitivity assay

Thirty stored positive serum samples from echinococcosis patients were tested (Table S3). Among them, 27 tested positive and 3 were negative, corresponding to a positive detection rate of 90%, indicating favorable diagnostic sensitivity of the method. Analytical sensitivity was further assessed (Table S3). Antibody dilution titers were determined for the three echinococcosis-positive serum samples, and the dilution titer was found to be 1:16, demonstrating satisfactory analytical sensitivity.

#### Repeatability assay

The repeatability results are presented in Table S4. The intra-assay CV ranged from 2.74% to 7.47% (<10%), and the inter-assay CV ranged from 6.02% to 9.74% (<15%), demonstrating excellent repeatability of the method.

#### Concordance rate assay

A head-to-head comparison was performed on 47 sheep serum samples using the blocking ELISA and the commercial Sheep Hydatid Cyst Eg95 Detection Kit (Shenzhen Kangbaide Biotechnology Co., Ltd.) (Table S5), the commercial kit identified 31 positive and 16 negative samples, whereas the blocking ELISA detected 28 positive and 19 negative samples. Statistical analysis revealed an overall concordance of 93.62% between the two assays.

### Serological detection

The ELISA results revealed that samples from the middle and late stages of infection tested positive in addition to an early-stage seropositivity rate of 66.67%. Consequently, the blocking ELISA established in this study successfully detected 8 out of 9 infected serum samples, yielding an overall positive detection rate of 88.89% (Table S6).

## DISCUSSION

Currently, recombinant protein expression systems are primarily divided into prokaryotic and eukaryotic systems (24). *E. coli* serves as the canonical prokaryotic expression host, whereas eukaryotic systems comprise mammalian cells, yeast, and insect cells (25). Owing to its well-defined genetic background, high expression efficiency, and simple manipulation, the *E. coli* prokaryotic system is extensively used for the large-scale production of recombinant proteins (26–28). In the present study, the pET-28a prokaryotic expression system was employed for Em18 expression, and efficient soluble expression of the target protein was attained by optimizing induction conditions. Previous studies have indicated that low-temperature induction (15°C) improves the yield of the fusion antigen rAgB8/1-Em18-Eg95 in prokaryotic hosts (29), which is consistent with the optimal conditions determined in this work. The underlying mechanism may be ascribed to reduced hydrophobic aggregation, enhanced protein stability, and promoted proper folding at low temperatures, thus partially alleviating protein degradation (30, 31). Moreover, low-temperature cultivation upregulates the expression of molecular chaperones in *E. coli*, which further promotes protein yield and activity (32–34). Accordingly, optimization of the induction temperature is crucial for generating rEm18 protein with desirable immunoreactivity.

IgG1 can mediate antibody-dependent cellular cytotoxicity (ADCC), antibody-dependent cellular phagocytosis (ADCP), and complement-dependent cytotoxicity (CDC) (35) and is, thus, considered a pivotal antibody isotype involved in protective immunity against parasitic infections. All six mAbs produced in this study were of the IgG1 heavy-chain isotype, in agreement with previous findings that IgG1 constitutes the dominant antibody subclass targeting Em18 in AE patients (36). This result implies that the humoral immune response elicited by Em18 may display a distinct isotype bias. Earlier studies have shown that IgG1 detection via ELISA offers superior diagnostic performance relative to total IgG and IgG2-IgG4 (37), providing a basis for IgG1-based serological surveillance in echinococcosis-endemic areas (38). Furthermore, antibody isotype identification offers a rational reference for the selection of purification resins. Compared with Protein A, Protein G exhibits higher binding specificity and affinity toward IgG1 (39), which can effectively diminish nonspecific impurity binding and enhance purification efficiency (40).

MAbs do not recognize the entire antigen molecule, but rather specific domains termed B-cell epitopes (41), the fundamental structural units responsible for eliciting humoral immune responses. Identification of antibody-recognized epitopes is critical for clarifying antigen-antibody interactions and developing improved diagnostic reagents (42). The epitope targeted by the six mAbs in this study was highly consistent with the predictions from previous studies (43, 44). Amino acid sequence alignment demonstrated that this epitope is highly conserved across *Echinococcus* species, with no homologous sequences detected in other taeniid cestodes or common parasites. These results confirm its high species specificity, which represents a distinct advantage for minimizing cross-reactivity in serological tests.

During HRP labeling of the selected blocking mAb 11E8C, the temperature and reaction time for HRP activation and antibody conjugation were strictly controlled to improve labeling efficiency. Following labeling, the conjugates were aliquoted and stored properly to minimize the effects of polymerization and inactivation on assay performance, thereby supporting the establishment of a method with favorable sensitivity, specificity, and reproducibility (45). The false-negative results observed in the concordance assessment of the blocking ELISA may be attributed to multiple factors, including sample quality, reaction conditions, and inherent methodological limitations (46, 47).

Notably, one early-infected sample presented a false-negative outcome in our clinical validation, which can be rationally interpreted by the serological window period of parasitic infection. At the early stage of pathogen invasion, antigen-specific IgG antibodies fail to be adequately synthesized and accumulated in peripheral blood, and their concentrations cannot reach the cutoff value of our established blocking ELISA, thereby leading to missed diagnosis of patients with early echinococcosis. As the primary immunoglobulin secreted upon acute infection, IgM can be detected considerably earlier than IgG in serum specimens. Accordingly, supplementary IgM antibody testing is capable of substantially improving the detection sensitivity for patients in the early phase of echinococcosis.

This study provides novel technical support for the serodiagnosis of echinococcosis by generating highly specific anti-Em18 mAbs, identifying a conserved B-cell epitope and establishing a well-performing blocking ELISA assay. Nevertheless, the present study has certain limitations, as the sample size for clinical validation was relatively small owing to restrictions on clinical serum collection and lacked validation with human sera as well as samples from different geographical regions and various infection stages. Additionally, the specific causes of false-negative results have not been elucidated, and the development of a standardized kit for this detection method remains unaccomplished. For future research, we will further expand the sample size of clinical specimens from diverse sources and different infection stages for comprehensive validation, advance the development and optimization of a blocking ELISA detection kit, and conduct in-depth investigations into the underlying causes of false-negative results to improve the assay system in a targeted manner. These efforts are expected to facilitate the practical application of this technology in the epidemiological screening and clinical diagnosis of echinococcosis.

### Conclusion

In summary, this study successfully achieved high-yield expression and purification of the *E. multilocularis* Em18 protein and generated six novel mAbs with excellent specificity. A comprehensive functional characterization was performed, including analyses of antigen specificity, antibody titers, isotype identification, and epitope mapping. Notably, evaluation using clinical samples demonstrated that the mAb-based blocking ELISA holds strong potential for the preliminary detection of Em18 antibody levels. These findings provide a critical foundation for the development of early and rapid diagnostic kits for echinococcosis.

## Data Availability

Data will be made available upon request.

## SUPPLEMENTAL MATERIAL

Fig. S1 spectrum.03933-25-s0001.tif Optimization of rEm18 expression and purification.

Fig. S2 spectrum.03933-25-s0002.tif Standard curve for determining Em18 protein concentration by BCA method.

Fig. S3 spectrum.03933-25-s0003.tif Purification of six anti-Em18 mAbs.

Supplemental tables spectrum.03933-25-s0004.docx Tables S1 to S6.

ASM does not own the copyrights to Supplemental Material that may be linked to, or accessed through, an article. The authors have granted ASM a non-exclusive, world-wide license to publish the Supplemental Material files. Please contact the corresponding author directly for reuse.
