# Supplementary material for: The establishment of a universal blocking ELISA for echinococcosis using immunoglobulin against a new conserved linear B-cell epitope
Source: Microbiol Spectr. 2026 Aug 19;14(10):e03933-25. doi: 10.1128/spectrum.03933-25 (PMC13689459; doi:10.1128/spectrum.03933-25)
Supplement: Supplementary file 1 [file spectrum.03933-25-s0004.docx]

**S1 Table** ELISA titers of six mAbs against Em18 protein (OD450).

| **Serum dilution** | **mAbs** | | | | | | | | | | **Negative control** |
| --- | --- | --- | --- | --- | --- | --- | --- | --- | --- | --- | --- |
|  | **11E2E** | **11E7C** | | **11E7E** | | **11E8C** | | **11E10D** | **8G6F** | |  |
| 1:2000 | 1.3493 | 1.3491 | 1.1399 | | 1.5300 | | 1.4710 | | 1.1339 |  | 0.2253 |
| 1:4000 | 1.1549 | 1.1817 | 0.8372 | | 1.4709 | | 1.3349 | | 0.7543 |  | 0.1300 |
| 1:8000 | 1.0350 | 1.0135 | 0.5732 | | 0.8821 | | 1.0087 | | 0.5110 |  | 0.1126 |
| 1:16000 | 0.7768 | 0.5192 | 0.3646 | | 0.6334 | | 0.7479 | | 0.3348 |  | 0.1027 |
| 1:32000 | 0.5234 | 0.3542 | 0.3469 | | 0.5412 | | 0.5058 | | 0.2477 |  | 0.1007 |
| 1:64000 | 0.3288 | 0.2666 | 0.2728 | | 0.2691 | | 0.3069 | | 0.2246 |  | 0.0942 |
| 1:128000 | 0.1900 | 0.2180 | 0.1219 | | 0.1752 | | 0.2545 | | 0.1571 |  | 0.0690 |
| 1:256000 | 0.1495 | 0.1773 | 0.1161 | | 0.1542 | | 0.1317 | | 0.1478 |  | 0.0689 |
| 1:512000 | 0.0991 | 0.1406 | 0.1122 | | 0.1324 | | 0.1188 | | 0.1108 |  | 0.0676 |
| 1:1024000 | 0.0956 | 0.0999 | 0.0868 | | 0.1282 | | 0.0861 | | 0.1045 |  | 0.0649 |

**S2 Table** Determination of blocking effects of 6 monoclonal antibodies

| **mAbs** | **OD450 nm** | | **PI (%)** |
| --- | --- | --- | --- |
|  | **+** | **−** |  |
| 11E2E | 0.1075 | 0.2260 | 52.4 |
| 11E7C | 0.1151 | 0.5121 | 77.5 |
| 11E7E | 0.0794 | 0.1755 | 54.8 |
| 11E8C | 0.1039 | 0.5132 | **79.8** |
| 11E10D | 0.0844 | 0.1929 | 56.3 |
| 8G6F | 0.1028 | 0.2585 | 60.2 |

**S3-1 Table** Diagnostic sensitivity test sesults of blocking ELISA

| **Serum ID** | **PI (%)** | **Serum ID** | **PI (%)** | **Serum ID** | **PI (%)** |
| --- | --- | --- | --- | --- | --- |
| 1# | 34.08 | 11# | 32.59 | 21# | 29.76 |
| 2# | 29.56 | 12# | 31.42 | 22# | 35.26 |
| 3# | 36.12 | 13# | 29.67 | 23# | 30.94 |
| 4# | 30.82 | 14# | 27.28 | 24# | 28.31 |
| 5# | 32.28 | 15# | 34.19 | 25# | 37.23 |
| 6# | 27.62 | 16# | 13.02 | 26# | 29.06 |
| 7# | 16.25 | 17# | 31.28 | 27# | 33.92 |
| 8# | 34.59 | 18# | 28.89 | 28# | 12.93 |
| 9# | 27.18 | 19# | 32.90 | 29# | 34.46 |
| 10# | 31.01 | 20# | 24.66 | 30# | 29.76 |

**S3-2 Table** Analytical sensitivity test results of blocking ELISA

| **Serum dilution** | **Sample ID/ PI (%)** | | |
| --- | --- | --- | --- |
|  | **1#** | **2#** | **3#** |
| neat | 79.12 | 70.38 | 71.82 |
| 1:1 | 77.49 | 69.00 | 70.80 |
| 1:2 | 72.40 | 64.92 | 66.86 |
| 1:4 | 64.33 | 57.97 | 61.08 |
| 1:8 | 42.69 | 42.91 | 37.97 |
| 1:16 | 25.01 | 32.59 | 30.30 |

**S4-1 Table** Intra-assay repeatability of the universal blocking ELISA.

| **Sample** | **PI of in-batch repeatability assay** | | | | | | | | | | |
| --- | --- | --- | --- | --- | --- | --- | --- | --- | --- | --- | --- |
|  | **M1** | **M2** | **M3** | **M4** | **M5** | **M6** | **M7** | **M8** | **M** | **SD** | **CV (%)** |
| 1# | 35.52 | 36.81 | 37.24 | 35.91 | 36.04 | 37.47 | 36.72 | 35.73 | 36.43 | 1.00 | 2.74 |
| 2# | 45.11 | 47.19 | 46.02 | 46.78 | 45.82 | 47.93 | 46.46 | 47.01 | 46.54 | 1.29 | 2.77 |
| 3# | 55.14 | 56.99 | 56.46 | 55.83 | 57.95 | 55.63 | 56.84 | 55.96 | 56.40 | 1.65 | 2.93 |
| 4# | 16.29 | 18.48 | 16.52 | 18.31 | 16.67 | 18.73 | 17.09 | 17.91 | 17.70 | 1.19 | 6.80 |
| 5# | 15.82 | 16.59 | 18.66 | 16.07 | 18.33 | 18.84 | 16.90 | 16.99 | 17.28 | 1.29 | 7.47 |
| 6# | 16.02 | 17.18 | 15.81 | 16.52 | 16.91 | 17.58 | 15.92 | 17.38 | 16.79 | 1.06 | 6.31 |

Note: 1#-3# are positive sera, 4#-6# are negative sera

### **S4-2 Table** Inter-batch repeatability of the universal blocking ELISA.

| **Sample** | **PI of batch-to-batch repeatability assay** | | | | | |
| --- | --- | --- | --- | --- | --- | --- |
|  | **Day 1**  **(Plates 1-5)** | **Day 2**  **(Plates 6-10)** | **Day 3**  **(Plates 11-15)** | **N** | **SD** | **CV (%)** |
| 1# | 33.86 | 38.12 | 31.55 | 34.51 | 3.36 | 9.74 |
| 2# | 39.21 | 43.56 | 37.02 | 39.93 | 3.34 | 8.36 |
| 3# | 56.88 | 61.02 | 54.15 | 57.35 | 3.45 | 6.02 |
| 4# | 15.82 | 14.18 | 16.76 | 15.59 | 1.32 | 8.47 |
| 5# | 16.35 | 14.66 | 17.22 | 16.08 | 1.31 | 8.15 |
| 6# | 16.62 | 15.02 | 17.35 | 16.33 | 1.17 | 7.16 |

Note: 1#-3# are positive sera, 4#-6# are negative sera

**S5 Table** Concordance test results between blocking ELISA and the commercial kit

| **Commercial kit** | **Blocking ELISA** | | | |
| --- | --- | --- | --- | --- |
|  | **Positive** | **Negative** | **Total** | **Coincidence rate** |
| **Positive** | 28 | 3 | 31 | 90.32% |
| **Negative** | 0 | 16 | 16 | 100.00% |
| **Total** | 28 | 19 | 47 | 93.62% |

**S6 Table** Results of blocking ELISA for mouse serum at different stages of infection

| Group | Number of Samples Tested | Number of Positive Samples | Positivity Rate(%) |
| --- | --- | --- | --- |
| WT-1 month post-infection (early) | 3 | 2 | 66.67 |
| WT-2 month post-infection (mid)) | 3 | 3 | 100.00 |
| WT-6 month post-infection (late)) | 3 | 3 | 100.00 |
| Total | 9 | 8 | 88.89 |

**Supplemental Figure and figure legends**

**Fig. S1.** Optimization of rEm18 expression and purification. The recombinant protein was expressed using the pET-28a prokaryotic system and purified by nickel-affinity chromatography, following systematic optimization of key induction parameters including temperature, duration, and IPTG concentration. (A)-(C) Temperatures (16 ℃, 25 ℃, 37 ℃) and durations (12 h, 14 h, 16 h, 20 h) analysis. M: Protein molecular weight marker; Lanes 1-4: Whole-cell lysates (12-20 h induction); Lanes 5-8: Corresponding soluble fractions; Lanes 9-12: Corresponding insoluble pellets; Lane 13: Non-induced control. (D) IPTG concentration optimization (0.2-1.0 mM IPTG). M: Protein molecular weight marker; Lanes 1-5: Soluble fractions; Lane 6: Non-induced control. (E) Affinity purification of rEm18. M: Protein molecular weight marker; Lanes 1-3: Input fractions (whole-cell lysate, soluble, and insoluble); Lane 4: Flow-through; Lane 5: Wash fraction with 40 mM imidazole; Lane 6-10: Gradient elution (100-500 mM imidazole); Lane 11: pET28a-BL21(DE3) bacterial lysate. (F) Densitometric analysis of soluble rEm18 under varied conditions.

**Fig. S2.** Standard curve for determining Em18 protein concentration by BCA method.

**Fig. S3.** Purification of six anti-Em18 mAbs.
